# Annotations

**Published:** 1902-04-12

**Authors:** 


### Annotations.

### Medical Advertising.

A workman who possesses but one tool which he applies to all sorts of uses is apt to take off the fineness of its edge, and to lessen its fitness for its original purpose, which is just what the General Medical Council appears likely to do with the single weapon by which it can defend its Register. For the punishment of medical men of all degrees, whatever their sins or peccadilloes, the Council has but one penalty, namely, expulsion from the Register, and there is but one offence for which this penalty may be inflicted, namely, "infamous conduct." Hence those who wish to extend the powers of the Council are very anxious to extend the meaning of the word "infamous" so as to make it applicable to what may be termed minor professional misdeeds, and more particularly to the crime of advertising. We cannot but fear, however, that thus to attach Pickwickian meanings to grave terms of reproach must materially blunt their force. We may quite agree with the *British Medical Journal* when it says that "the rule against advertising is one of the oldest and most salutary of those binding on the medical profession," but advertising is of many degrees, and when we are asked to apply the term "infamous" to this offence, and when its use for this purpose is excused on the ground that it is not a term chosen by the Council but is "employed in the Medical Act to designate those offences which, while they may involve no breach of the ordinary law, yet render the offender unfit to hold a place on the Medical Register," and when we are further told that it is a "technical term" signifying "conduct unworthy of a registered medical practitioner," we can only ask where does it find all this in the Medical Act? So to extend the significance of the word "infamous" as to include under it those who advertise is to take away from it all point and meaning. Men advertise themselves in a multitude of ways—by means of books, by medical bulletins, by notices, and even by portraits in the society journals, by brass plates or red lamps, or by the hateful, but we presume effective, handbill. It is impossible to draw the line, for these are but different methods appealing to different classes. To apply the term "infamous" to a crime of so many degrees, some of which might, we are afraid, be brought home to men whom the world receives with open arms would be a very doubtful proceeding, sure to end by taking off the edge of the one weapon which the Council possesses for the punishment of serious delinquencies.

### The Tenure of Hospital Appointments.

The report of the proceedings at the annual meeting of the Torbay Hospital, which we publish in another column, serves well to illustrate the evils connected with what we cannot but describe as the worst possible mode of appointing medical officers to a hospital, namely by annual election. So far as we gather from the report the rule at this hospital provides that the medical officers shall be elected by a special committee appointed for that purpose, which is a very proper course, but—and here is the weak spot—that their retention of their appointments shall be subject to their annual re-election. Nothing could be more calculated to keep a hospital constantly in a ferment of unrest, and to prevent that careful and quiet attention to the wants of the sick which is essential to its smooth working, than this arrangement, according to which, if a man would retain his appointment, he must perforce keep himself in touch with all the outside intrigues for his displacement from office which such an uncertain tenure will certainly provoke. All appointments on the honorary staff of a hospital ought to be made by a special committee appointed for that purpose only, and largely but not exclusively composed of office bearers, such as president, treasurer, etc., and of members of the board of management. This committee ought not to be appointed *en bloc*, but a certain proportion of its members should retire every year so that its constitution shall never even seem to be affected by any prospective election. In this way there is every likelihood of the best and most suitable man being elected. But when once appointed he certainly ought, if he is to do his best for the institution, to be free from all care as to his tenure of office. If the governors of a hospital choose to insert a rule to the effect that a medical officer shall be liable to be dismissed from office by a General Board called for that purpose, by motion made and seconded and of which notice has been given, we would make no objection. Such a provision might in some extreme cases get a hospital out of a difficulty. It would, however, be very seldom put into operation, and never without ample notice. But to leave it open to any little clique in a town to organise a scheme according to which, without notice and without the statement of any reason, the name of a tried and trusted official might be passed over in the more or less formal re-election of officers, and the name of some outsider quietly substituted, is a plan which must inevitably keep an institution in a continual ferment of suspicion and unrest, and must be injurious to the well-being of all concerned. Any rule which allows such a condition of affairs can only be described as a thoroughly bad one.

### An Awkward Problem.

What should a doctor do if he finds a patient in his surgery suffering from small-pox? A medical man, practising at Tottenham, found himself in this awkward position. According to his evidence, at a time when he had twenty people in his surgery, a patient came in who proved to be suffering from small-pox, and what he did was to write a notification certificate and send the patient home, telling him to leave this certificate in the letter-box of the Medical Officer of Health, thinking that this was the quickest way of conveying the notification. The patient, however, seems to have walked into the latter gentleman's surgery where there were several patients, and delivered the certificate *in propriâ personâ.* Hence the prosecution of the doctor for "aiding and abetting and procuring" the said patient "to expose himself while suffering from small-pox," it being held that to send the certificate by the infected person was to send out an infected document. The summons was dismissed. There is no doubt, however, that the proper course would have been to tell the patient to avoid everyone and go straight home; to send immediate notification to the Medical Officer of Health; and to take steps to secure the revaccination of all the patients in the surgery.

